# Glioblastoma misdiagnosed as acute disseminated encephalomyelitis following coronavirus disease vaccination

**DOI:** 10.1590/0037-8682-0400-2022

**Published:** 2022-12-16

**Authors:** Alptekin Tosun, Merve Nur Tasdemir

**Affiliations:** 1 Giresun University, Faculty of Medicine, Department of Radiology, Giresun, Turkey.

In recent years, coronavirus disease (COVID-19) has been a leading cause of death worldwide^1^. Acute disseminated encephalomyelitis (ADEM) and Guillain-Barré syndrome were observed following COVID-19 vaccinations^2^.

**FIGURE 1:**
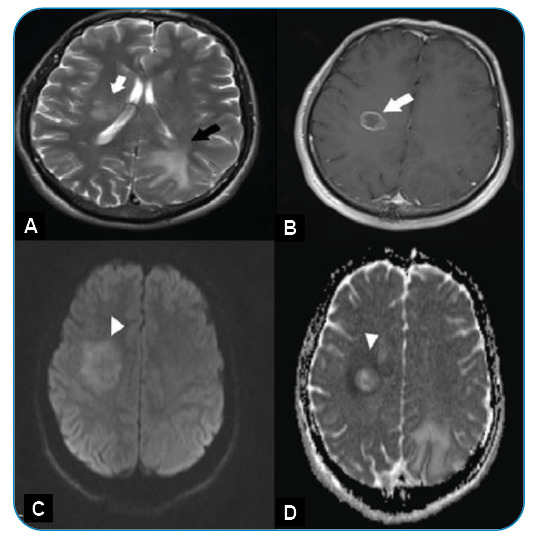
**(A)** The axial T2-weighted image demonstrates a heterogeneous lesion with indistinct borders in the right frontal lobe (white arrow) and vasogenic edema in the left parieto-occipital white matter without a mass (black arrow). **(B)** The contrast-enhanced axial T1-weighted image shows mild peripheral contrast enhancement (white arrow). **(C,D)** The diffusion-weighted image and apparent diffusion coefficient map reveal peripheral restricted diffusion (arrow head).

**FIGURE 2:**
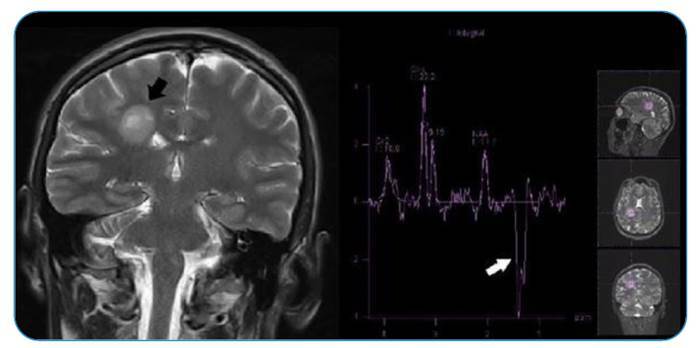
MRI spectroscopy of the right frontal lobe lesion (black arrow) exhibits increased choline and decreased NAA ratios with lipid and lactate peaks (white arrow).

**FIGURE 3:**
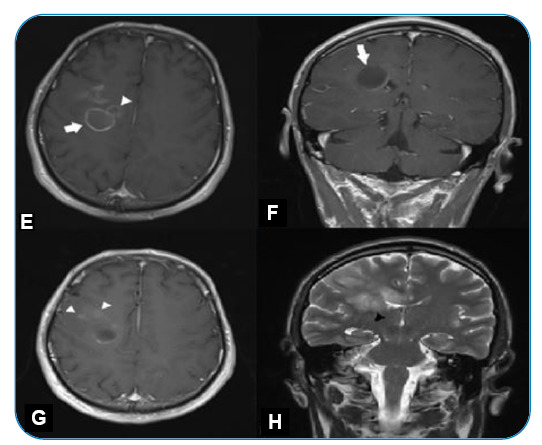
**(E,F,G)** On day 10, control axial and coronal T1-weighted contrast-enhanced images show a circular contrast enhancement in the cystic lesion (white arrow) and mild heterogeneous enhancement of satellite lesions with indistinct borders extending to the frontal cortex (white arrow heads). **(H)** Coronal T2-weighted images show increased size of the widest lesion (black arrowhead) extending to the corpus callosum.

A 40-year-old man presented with left hemiparesis. He had received COVID-19 vaccination 3 weeks before. Brain magnetic resonance imaging (MRI) showed a central cystic necrotic lesion with indistinct borders in the right frontal lobe as mild peripheral contrast enhancement surrounded by smaller nodular lesions. The right frontal lobe cortex was edematous. Vasogenic edema was observed in the left parieto-occipital lobe without a mass (Figure 1). MRI spectroscopy revealed elevated choline and reduced N-acetyl aspartate (NAA) metabolite ratios coexisting with lactate and lipid peaks, consistent with inflammatory conditions instead of tumors (Figure 2). For 5 days, 30 mg/kg/day of methylprednisolone was administered. MRI performed on day 10 showed increased size of the widest lesions. A circular enhancement pattern is found instead of the visible heterogeneous enhancement pattern at the center of the lesion (Figure 3). MRI perfusion showed increased cerebral blood flow and volume in the centrum semiovale extending toward the corpus callosum. A biopsy was performed, and the patient was diagnosed with grade IV glioblastoma.

The adverse effects of vaccines, particularly during pandemics, may be exaggerated and lead to misdiagnoses. Even with a history of vaccination, we should consider a tumor in the differential diagnosis.
